# Cep55 regulates spindle organization and cell cycle progression in meiotic oocyte

**DOI:** 10.1038/srep16978

**Published:** 2015-11-19

**Authors:** Zhao-Yang Xu, Xue-Shan Ma, Shu-Tao Qi, Zhen-Bo Wang, Lei Guo, Heide Schatten, Qing-Yuan Sun, Ying-Pu Sun

**Affiliations:** 1 The Reproductive Medical Center, the First Affiliated Hospital, Zhengzhou University, Zhengzhou, 450052 China; 2 State Key Laboratory of Reproductive Biology, Institute of Zoology, Chinese Academy of Sciences, Beijing, 100020 China; 3 Department of Veterinary Pathobiology, University of Missouri-Columbia, MO 65211, USA

## Abstract

Cep55 is a relatively novel member of the centrosomal protein family. Here, we show that Cep55 is expressed in mouse oocytes from the germinal vesicle (GV) to metaphase II (MII) stages. Immuostaining and confocal microscopy as well as time lapse live imaging after injection of mRNA encoding fusion protein of Cep55 and GFP identified that Cep55 was localized to the meiotic spindle, especially to the spindle poles at metaphase, while it was concentrated at the midbody in telophase in meiotic oocytes. Knockdown of Cep55 by specific siRNA injection caused the dissociation of γ-tubulin from the spindle poles, resulting in severely defective spindles and misaligned chromosomes, leading to metaphase I arrest and failure of first polar body (PB1) extrusion. Correspondingly, cyclin B accumulation and spindle assembly checkpoint (SAC) activation were observed in Cep55 knockdown oocytes. Our results suggest that Cep55 may act as an MTOC-associated protein regulating spindle organization, and thus cell cycle progression during mouse oocyte meiotic maturation.

To investigate early development in mammalian species, Edwards *et al.* developed the method of oocyte *in vitro* maturation (IVM)^1^,^2^. With the development of assisted reproductive techniques, IVM has been increasingly used in the past two decades in order to eliminate or reduce drug costs and side effects associated with controlled ovarian stimulation. Oocyte maturation is a complex and precisely synchronized process affected by many factors. Oocyte maturation refers to the meiotic process that takes place from the germinal vesicle (GV) stage to the metaphase II stage. The first indication for this process is the disappearance of the germinal vesicle as observed under the light microscope, which is called germinal vesicle breakdown (GVBD). After GVBD, oocytes pass through metaphase of the first meiotic division, followed by the first polar body extrusion and entry into the second meiotic division. Thereafter, oocytes are arrested at metaphase of the second meiotic division until fertilization takes place^3^.

Cell division involves precise spindle organization and chromosome segregation. In mitosis, this process significantly depends on centrosomes. Centrosomes are well known for their role in microtubule nucleation. The centrosome functions as microtubule organizing center (MTOC) to regulate spindle formation and to ensure the proper segregation of chromosomes. Typical centrosomes as best known for somatic cells are absent in mammalian meiotic oocytes^4^; instead, oocytes use MTOCs that contain centrosomal proteins without containing centrioles to accomplish meiotic spindle organization^5^.

Centrosomal proteins function in several ways to control cell division in mitosis^6^. They control, centriole duplication, spindle organization, spindle-kinetochore assembly, cell polarity and cell cycle checkpoint signal mechanisms, and cell cycle progression. Several studies also demonstrated the centrosome’s role in cytokinesis^7^,^8^. Cells without centrosomes failed to accomplish cytokinesis, despite the formation of a cleavage furrow. Correspondingly, several centrosome-associated proteins localize first to the centrosomes, and then migrate to the midbody in telophase, where they play an important role in the process of cytokinesis^9^,^10^,^11^,^12^.

Among the centrosomal proteins, Cep55, a coiled-coil protein whose gene is located in 10q23.33, is important for the completion of cytokinesis. It is located to the centrosomes, mitotic spindle, spindle midzone and midbody in somatic cells^12^,^13^,^14^. It recruits two components of the ESCRT machinery: the ESCRT-I complex and ALIX^15^,^16^, leading to the recruitment of ESCRT-III subunits, which are required for normal midbody morphology and are believed to have membrane scission activity^17^,^18^,^19^. In general, Cep55 is important for midbody integrity and cell abscission in cytokinesis^12^,^14^,^20^,^21^,^22^. Here we show that Cep55 functions differently in meiotic oocytes than in mitotic cells.

## Results

### Expression and subcellular localization of Cep55 during mouse oocyte meiotic maturation

**Figure 1:**
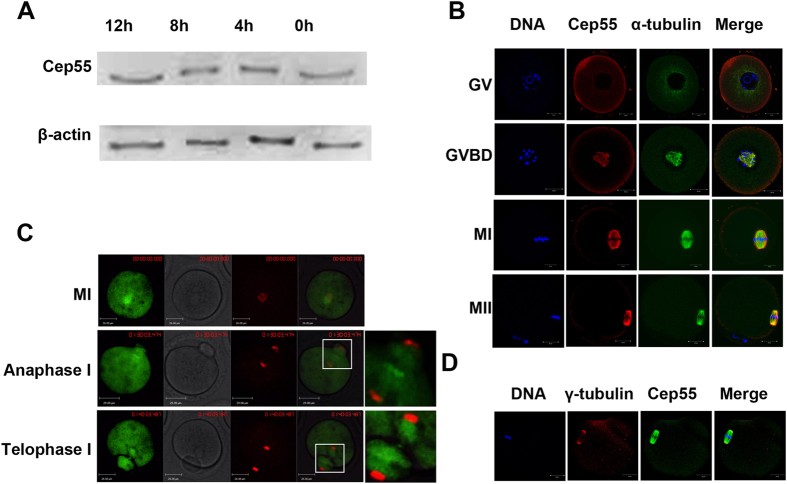
Expression and subcellular localization of Cep55 during mouse oocyte meiotic maturation. (**A**) Expression level of Cep55 identified by Western blotting. A total of 200 oocytes were collected after culture for 0, 4, 8, and 12 h, representing GV, GVBD, MI and MII stages, respectively. The molecular weight of Cep55 and β-actin were 55 kD and 43 kD, respectively. (**B**) Subcellular localization of Cep55 as revealed by immunofluorescent staining and confocal microscopy. Oocytes at the four stages were stained with antibody against Cep55 (red) and α-tubulin (green); Each sample was stained with Hoechst 33342 to visualize DNA (blue). Bar = 20 μm. (**C**) Screen shot of live cell imaging of Cep55 localization. mRNA encoding fusion protein of Cep55 and GFP (green) was injected into the oocytes to trace the dynamics of Cep55 from MI to telophase I stages. Hoechst 33342 (red) was added to the culture medium 15 min before the oocytes were placed on the microscope stage. (**D**). Co-localization of γ-tubulin and Cep55 at spindle poles at the MII stage. Oocytes cultured for 12 h (MII) were stained for γ-tubulin (red), Cep55 (green) and DNA (blue). Bar = 20 μm.

Oocytes were cultured for 0 h, 4 h, 8 h and 12 h, representing the germinal vesicle (GV), prometaphase I, metaphase I and metaphase II stages, respectively. Western blot showed that Cep55 protein was expressed from GV to metaphase II stages, without detectable change (Fig. 1A). Both immunostaining and confocal microscopy as well as live cell imaging after intra-oocyte injection of mRNA encoding fusion protein of Cep55 and GFP showed that Cep55 dispersed in the cytoplasm of GV stage oocytes. After germinal vesicle breakdown (GVBD), Cep55 gradually accumulated around the nuclear area, and it then co-localized with α-tubulin of the spindle. In metaphase, it was found to distribute on spindle microtubules and accumulate at the spindle poles, while it was located at the midzone in anaphase and at the midbody in telophase (Fig. 1B–D). We found that it is hard to localize Cep55 at the midbody by immunostaining of fixed samples in both mouse and human mitotic cells and meiotic oocytes (Supplemental Fig. 1), but it did show a transient localization at the midbody when observed by time-lapse live imaging (Supplemental video 1 and 2).

### Knockdown of Cep55 causes abnormal spindle and misaligned chromosomes in meiotic oocytes

**Figure 2:**
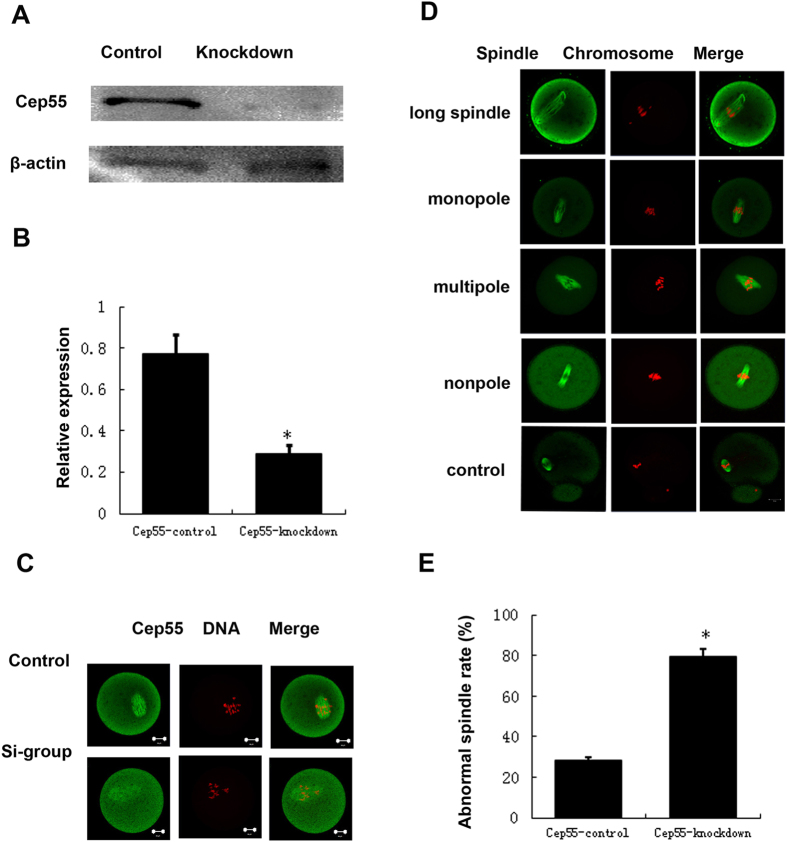
Depletion of Cep55 causes severely abnormal spindle and misaligned chromosomes in oocytes. (**A**) Western blotting showing knockdown of Cep55 protein after siRNA. After microinjection of Cep55 or control siRNA, the oocytes were incubated in M16 medium containing 200 μM IBMX for 24 h, then washed 9 times and placed in IBMX-free M16 medium for 6 h, followed by western blotting. The molecular weights of Cep55 and beta-actin are 55 kD and 43 kD, respectively. A total of 200 oocytes were used in each lane. (**B**) Relative expression of Cep55 after siRNA treatment. Data are presented as means + -SEM of 3 independent experiments (p < 0.05). (**C**) Confocal image showing knockdown of Cep55 protein after siRNA. After microinjection of Cep55 or control siRNA, the oocytes were incubated in M16 medium containing 200 μM IBMX for 24 h, then washed 9 times and placed in IBMX-free M16 medium for 4 h. A total of 132 oocytes were assessed in the si-group and 143 oocytes were assessed in the control group. (**D**) After microinjection of Cep55 or control siRNA, the oocytes were incubated in M16 medium containing 200 μM IBMX for 24 h, then washed 9 times and placed in IBMX-free M16 medium for 14 h of culture, followed by immunostaining with α-tubulin (green) and Hoechst 33342 (red). In the Cep55-depletion group, the oocytes showed various kinds of severely abnormal spindles and misaligned chromosomes. Bar = 20 μm. (**E**) The rate of oocytes with abnormal spindles and misaligned chromosomes in the Cep55 depletion group and control group. Data are presented as means + -SEM of 4 independent experiments (*p < 0.05).

To study the roles of Cep55 in mouse oocyte meiotic maturation, we knocked down Cep55 by its specific siRNA injection. Both Western blotting and immunostaining showed that the expression level of Cep55 was notably reduced (Fig. 2A–C). In the Cep55 knockdown group, oocytes showed different kinds of severely abnormal spindles and misaligned chromosomes. The major spindle defects were elongated spindles and spindles with abnormal poles, including spindles with no apparent pole, one pole, or multi-poles; severe chromosome misalignment was also observed (Fig. 2D). The proportion of oocytes with abnormal spindles in Cep55 knockout oocytes was significantly higher than in the control group (P < 0.01, Fig. 2E). Among the abnormal spindles, the long spindle configuration accounted for 38.9%, the multipole spindle configuration accounted for 7.6%, and mono- and non-polar spindles represented the majority, accounting for 53.4%.

### Knockdown of Cep55 leads to the disassociation of γ-tubulin from spindle poles

**Figure 3:**
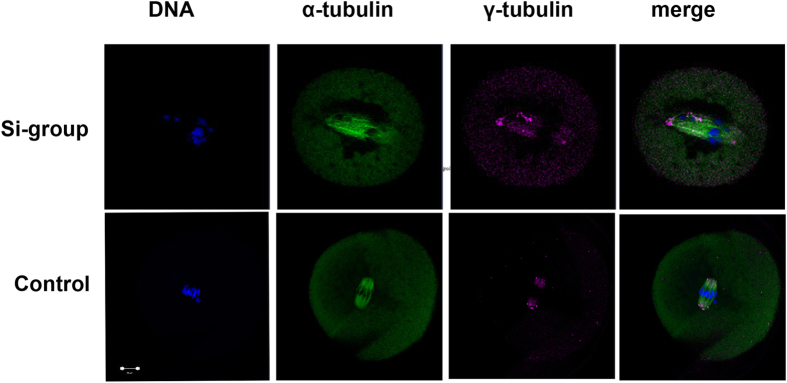
Knockdown of Cep55 causes disassociation of γ-tubulin from spindle poles. Oocytes injected with Cep55 siRNA or control siRNA were cultured in fresh M16 medium for 8 h after 24 h arrest at the GV stage by IBMX, followed by staining of α-tubulin (green), γ-tubulin (purple) and DNA (blue) in the control group; γ-tubulin was associated with the spindle poles at the MI stage, whereas in the Cep55 knockdown group, γ-tubulin dissociated from abnormal spindle poles. Bar = 20 μm. A total of 160 oocytes (47 + 52 + 61) were assessed in the si-group and 166 oocytes (57 + 57 + 52) were assessed in the control group.

It is well known that the γ-tubulin complex is the marker of MTOCs, with functions in microtubule nucleation and spindle formation during cell division. Since Cep55 partially co-localized with the γ-tubulin complex, we further investigated the effect of Cep55 depletion on γ-tubulin localization. As shown in Fig. 3, the depletion of Cep55 caused the dissociation of γ-tubulin from the disrupted spindle poles, leading to irregular localization of γ-tubulin along the spindle fibers or in the cytoplasm.

### Knockdown of Cep55 causes increased level of cyclin B1, MI arrest, and reduced PB1 extrusion

**Figure 4:**
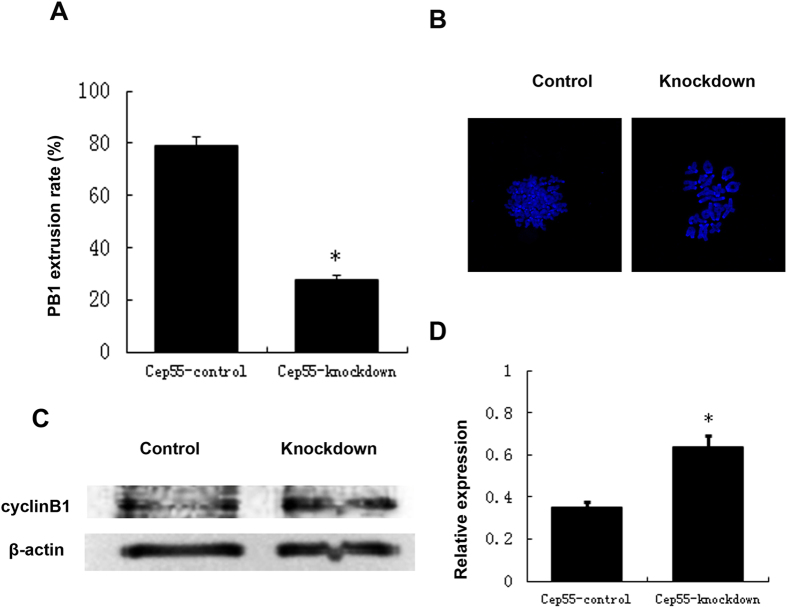
Cep55 knockdown causes increase of cyclin B1 level, non-segregation of homologous chromosomes, and failure of PB1 extrusion. After microinjection of Cep55 siRNA or control siRNA, the oocytes were placed in M16 containing IBMX for 24 h, then washed 8 times and transferred into IBMX-free M16 medium for 14 h to observe meiotic cell cycle progression. (**A**) Percentages of first polar body extrusion in the knockdown group and control group. Data are presented as means ± SEM of 4 independent experiments (*p < 0.01). (**B**) Failure of homologous chromosome segregation after Cep55 knockdown. The ratio of tetrads is 35/44 in the Cep55 knockdown and 7/41 in the control group. (**C**) Higher cyclin B1 level in Cep55 knockdown oocytes. Oocytes of the Cep55 knockdown group and control group were cultured in IBMX-free medium for 9.5 h, followed by western blotting. (**D**) The relative expression level of cyclin B1 after Cep55 knockdown. Data are presented as means + -SEM of 3 independent experiments (p < 0.05).

After microinjection of Cep55 specific siRNA or control siRNA, oocytes were placed in M16 medium containing 250 μM IBMX for 24 h. Oocytes were then cultured in IBMX-free M2 medium for 14 h. As shown in Fig. 4A, the PB1 extrusion rate in the Cep55-knockdown group was significantly lower than that in the control group. Most of the oocytes were blocked at the prometaphase/metaphase I stage, displaying abnormal spindles and misaligned chromosomes (Fig. 2D). Chromosome-spreading test confirmed failed homologous chromosome separation; all chromosomes were still tetrads even after 14 h of culture, while the control extruded the PB1 and separated the homologous chromosome (Fig. 4B). Western blot analysis showed that the expression level of cyclin B1 was much higher in the knockdown group compared to the control group at 9.5 h of culture in IBMX-free medium, suggesting the lack of degradation of cyclin B1 in the knockdown group (Fig. 4C,D), and thus failure of anaphase entry.

### Cep55 knockdown causes activation of the SAC

**Figure 5:**
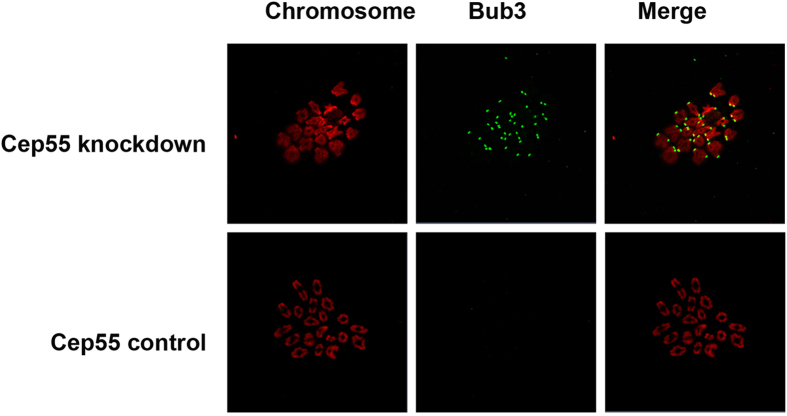
Cep55 knockdown caused activation of SAC in MI-arrested oocytes. After microinjection of Cep55 siRNA or control siRNA, the oocytes were placed in M16 containing IBMX for 24 h, then washed 9 times and transferred into IBMX-free M16 medium for 9.5 h. Bub3 (green) was localized at the kinetochores in the Cep55 si-group, while in the control group, Bub3 was dissociated from kinetochores of separating homologous chromosomes (red).

To further explore the reason for the MI arrest in Cep55 knockdown oocytes, the SAC protein Bub3 was immuno-stained. The result showed that Bub3 signal still existed on the chromosome kinetochores in the Cep55-knockdown oocytes after 9.5 h of culture, indicating activation of the SAC checkpoint. In contrast, the control oocytes entered anaphase, without detection of Bub3 on kinetochores (Fig. 5)

### Immunoblocking of Cep55 does not affect the PB1 extrusion

**Figure 6:**
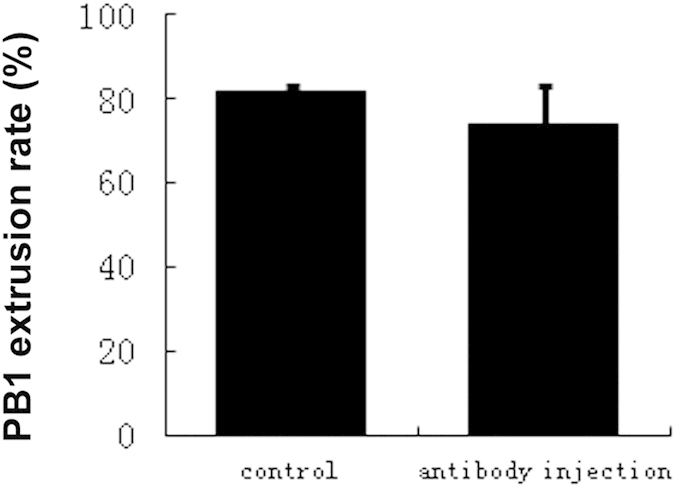
Cep55 immuno-blocking does not affect the PB1 extrusion rate in anaphase oocytes. Cep55 antibody was microinjected into oocytes after 9–9.5 h of culture, then transferred to M16 medium for 5 h of additional culture. Data are presented as means + -SEM of 3 independent experiments (p > 0.05).

To further clarify whether Cep55 functions in mouse oocyte cytokinesis, we microinjected Cep55 antibody at 9–9.5 h (corresponding to anaphase I) after culture and found no difference in the PB1 extrusion rate between the antibody injection and control groups (Fig. 6).

## Discussion

Mitosis and meiosis are different cellular processes. The regulation of meiotic spindle organization and chromosome segregation in the absence of typical centrosomes in mammalian oocytes has not been studied as well as that in mitotic somatic cells. Our study has shown that Cep55, a member of centrosomal proteins, plays an important role in spindle integrity and cell cycle progression in meiotic mouse oocytes. Cep55 locates to the spindle poles, binds to microtubules, and functions as major player of meiotic spindle assembly and chromosome segregation. Knockdown of Cep55 causes metaphase arrest and homologous chromosome segregation failure by causing spindle defects and SAC activation during first oocyte meiosis.

In our study, Cep55 shows different characteristics in meiotic oocytes compared to mitotic cells. In mitosis, Cep55 plays a role in cytokinesis by recruiting ESCRT proteins to the midbody. However, knockdown of Cep55 in meiotic oocytes lead to spindle organization defects and chromosome misalignment, arresting the cell cycle in the MI phase. These results suggest that the main function of Cep55 in meiosis is to organize microtubules to form the spindle. In mitosis, Cp55 was found to be involved in cytokinesis. However, we could not determine Cep55 functions in cytokinesis in Cep55 knock-down oocytes as is known for mitosis in somatic cells, since oocytes were arrested in metaphase I after Cep55 siRNA injection. To clarify whether Cep55 functions in oocyte meiotic cytokinesis, we performed immunoblocking of Cep55 by antibody injection in anaphase oocytes, and we did not find an effect on PB1 extrusion, suggesting that Cep55 might not play a role in cytokinesis in meiosis.

The centrosome consisting of centrioles and pericentriolar material was found to be the primary MTOC in animal cells^23^. In spite of their different shapes and sizes, all MTOCs depend on γ-tubulin to nucleate microtubules. γ-Tubulin is a highly conserved component of MTOCs and essential for microtubule organization^24^,^25^,^26^,^27^,^28^. γ-Tubulin was described as a part of a large complex of ring shape when examined with electron microscopy, subsequently termed γ-tubulin ring complex (γ-TuRC)^29^. The factors that influence proper recruitment of γ-tubulin to the spindle poles usually cause mitotic failure with defective spindles^30^. We found that the depletion of Cep55 lead to the dissociation of γ-tubulin from spindle poles, suggesting that Cep55 may play a role in spindle organization by participating in the association of the γ-tubulin ring complex with MTOCs.

Due to the severe spindle defects and chromosome misalignment, the oocytes were arrested at the MI stage as indicated by the failure of homologous chromosome segregation and first polar body emission. Cyclin B1 plays a key role in regulating the exit from the M-phase homologue disjunction was inhibited due to the stabilisation of cyclin B1^31^. Its degradation at the metaphase I-to-anaphase I transition by APC/C-Cdc20 causes a rapid decline in CDK1 activity, leading to exit from the M phase. In our study, the level of cyclin B1 in the Cep55-knockdown group was significantly higher than that in the control group at 9.5 h of culture, giving solid evidence that these oocytes are arrested in metaphase I.

SAC proteins including the Mad (mitotic-arrest deficient) proteins and Bub (budding uninhibited by Benz imidazole) proteins play critical roles in supervising proper chromosome segregation^32^,^33^. These proteins accumulate at kinetochores, blocking APC/C activity, preventing anaphase onset until all chromosomes accomplish equatorial alignment and bipolar attachments to the mitotic or meiotic spindle microtubules displaying proper tension. Bub3, a member of SAC, is part of the MCC in mammalian cells. Our previous study has shown that Bub3 plays an important role in regulating mouse oocyte meiotic anaphase entry^34^. In this study, Bub3 signals were detected at kinetochores in MI arrested oocytes in the Cep55 knockdown group after 9.5 h (even 12 h) of culture. However, the signals were absent in the control group at 9.5 h of culture, suggesting that in the knockdown group, MI arrest was due to the extended SAC activation caused by abnormal spindle and misaligned chromosomes.

In summary, our study shows that Cep55 plays an important role in spindle integrity, chromosome alignment, and thus metaphase-to-anaphase transition, but not in cytokinesis in meiotic oocytes, suggesting that the same protein may have different functions in meiosis and mitosis.

### Reagents

Rabbit polyclonal anti-Cep55 antibody was bought from Abcam (ab170414); mouse monoclonal anti-α tubulin antibody was purchased from Cell Signaling Technology (#3873); mouse monoclonal anti-γ-tubulin was obtained from Sigma-Aldrich Co (#T6557); rabbit polyclonal anti-bub3 antibody was obtained from Santa Cruz Biotechnology (sc-28258). FITC or TRITC conjugated goat anti-mouse IgG (H + L) and anti-rabbit IgG (H + L) were produced by Jackson Immnoresearch Laboratories, Inc. and sub-packaged by Zhongshan Golden Bridge Biotechnology Co. LTD (#ZF-0311, Zf-0316, Zf-0313, and Zf-0312). All other reagents were bought from Sigma Aldrich except when noted otherwise.

## Methods

### Mouse oocyte culture

The GV oocytes were collected from ovaries of 6–8 week-old female ICR mice in M16 medium (Sigma) supplemented with or without 100 μM IBMX. IBMX was used to prevent GVBD. Oocytes were placed in M16 medium under liquid paraffin oil at 37 °C in an atmosphere of 5% CO_2_ in air for specific time periods of further culture.

### SiRNA knockdown and antibody injection

SiRNAs were chemically synthesized by Gene Pharma. A volume of 2 mM siRNA (5′-CACCUAAGGUCAAGAUAUATT-3′; 5′-UAUAUCUUGACCUUAGGUGTT-3′) was microinjected into the oocyte to knockdown the Cep55 by keeping oocytes at the GV stage for 24 h in IBMX-containing medium. The same volume of negative control siRNA (5′-UUUUCCGAACGUGUCACGUTT -3′; 5′ACGUGACACGUUCGGAGAATT-3′) was also injected as control. Antibody injection was performed at 9–9.5 h of oocyte culture in M16 medium, and polar body emission was examined at 14h of culture.

### Immunofluorescence microscopy

Oocytes were fixed in 4% paraformaldehyde for 30 min, permeabilized with 0.5% Triton X-100 for 20 min, and blocked with 1% BSA for 24 h. Thereafter, oocytes were incubated with primary antibody at 4 °C for 24 h, and secondary antibody for 1 h at room temperature. Nuclei were labeled with Hoechst 33342. Cover slips were mounted with diazabicyclooctane antifade medium and visualized using Carl Zeiss LSM 780 confocal microscope.

For double staining of Cep55 and α/γ-tubulin, after incubation with secondary antibody of Cep55 and five washes in washing solution, oocytes were blocked again in 1% BSA for 1 h at room temperature, then stained with mouse polyclonal anti-α/γ-tubulin primary antibody overnight at 4 °C. After washing 5 times, they were incubated with FITC/CY5 conjuncted goat anti-mouse IgG for 1 h. Finally, oocytes were stained with Hoechst 33342 for 10 min.

### Chromosome spreading

Oocytes were placed in Tyrode’s solution (Sigma, T1788) to remove the zona pellucida. A 1 cm × 1 cm frame was drawn on a glass slide with a hydrophobic pen and 100 μl methanol: glacial acetic acid (3:1) was added in the frame area. Oocytes were dropped onto the slide for breaking and fixing. Then 10 μg/ml Hoechst 33342 was added to stain the chromosomes.

### Western blot

Protein extracts of 200 oocytes were used for polyacrylamide gel electrophoresis and separated proteins were transferred to nitrocellulose membranes. The membranes were blocked in 5% bovine serum albumin and then incubated with primary antibodies overnight at 4 °C. Thereafter, the membranes were incubated with secondary antibody for 1.5 h at room temperature. Western blot detection was performed with SuperSignal™ West Pico Chemiluminescent Substrate. The blots were visualized using a Chemi Doc Tm XRS + system (Bio-RAD).

### Cep55 expression plasmid construction and mRNA microinjection

Total RNA was extracted from 100 mouse GV oocytes using RNeasy micro purification kit (Qiagen) and the first strand cDNA was generated with cDNA synthesis kit (Takara), using poly (dT) primers. The nested PCR was used to amplify the full length of Cep55 cDNA. (Primers: F1: CGGTGCCACCCAAGTTAC, R1: CAAGGGCAGATGGAGTTTC; F2: CCCGCGGCCGCATGTCTTCAAGAAGTCCCAA, R2: GGGGTCGACCTTCATGCAGTATTCGACAT). The PCR products were digested using FseI and AscI (New England Biolabs, Inc.) and linked with GFP plasmid. The fusion plasmid was transfected to T1 competent cell (Transgen Biotech). Capped mRNA was produced by the mMESSAGE mMACHINE T3 (Ambion) with the linearized plasmids (linearized by Sfi, New England Biolabs, Inc), followed by tailing with poly (A) polymerase Tailing kit (Epicenter, AP-31220). Finally, an RNeasy cleanup kit (Qiagen) was used to purify the mRNA. The mRNA was microinjected into the oocyte cytoplasm for live cell imaging.

### Time-lapse live-cell imaging

Cep55 protein and chromosome dynamics were filmed on a Perkin Elmer precisely Ultra View Vox confocal imaging system. Exposure time was set ranging from 200–700 ms depending on the cep55-GFP fluorescence level. The acquisition of digital time-lapse image was controlled by Volocity software packages. Confocal images of spindles and chromosomes in live oocytes were acquired with a 20× objective on a spinning disk confocal microscope (Perkin Elmer).

### Statistical analysis

For each experiment, at least 3 replicates were performed. Statistical analyses were conducted by analysis of variance. Differences between two groups were compared by using an unpaired Student’s t-test, and p < 0.05 was considered to be significant.

### Ethic statement

All experimental protocols were approved by the Institute of Zoology, Chinese Academy of Sciences. All animal experiments were approved by the Animal Ethics Committee of Chinese Academy of Sciences and performed in accordance with the Committee guidelines of the Institute of Zoology, Chinese Academy of Sciences.

## Additional Information

**How to cite this article**: Xu, Z.-Y. *et al.* Cep55 regulates spindle organization and cell cycle progression in meiotic oocyte. *Sci. Rep.* **5**, 16978; doi: 10.1038/srep16978 (2015).

## Supplementary Material

Supplementary Information

Supplementary video 1

Supplementary video 2
