# Supplementary material for: Cep55 regulates spindle organization and cell cycle progression in meiotic oocyte
Source: Sci Rep. 2015 Nov 19;5:16978. doi: 10.1038/srep16978 (PMC4652202; doi:10.1038/srep16978)
Supplement: Supplementary Information [file srep16978-s1.doc]

**Cep55 regulates spindle organization and cell cycle progression in meiotic oocyte**

**Zhao-Yang Xu**^1^**, Xue-Shan Ma**^2^**, Shu-Tao Qi**^2^**, Zhen-Bo Wang**^2^**, Lei Guo**^2^**, Heide Schatten^3^, Qing-Yuan Sun**^2,*^**, Ying-Pu Sun**^1,*^

1. The Reproductive Medical Center, the First Affiliated Hospital, Zhengzhou University, Zhengzhou, 450052 China

2. State Key Laboratory of Reproductive Biology, Institute of Zoology, Chinese Academy of Sciences, Beijing, 100020 China

3. Department of Veterinary Pathobiology, University of Missouri-Columbia, MO 65211, USA

**Supplementary figure1**


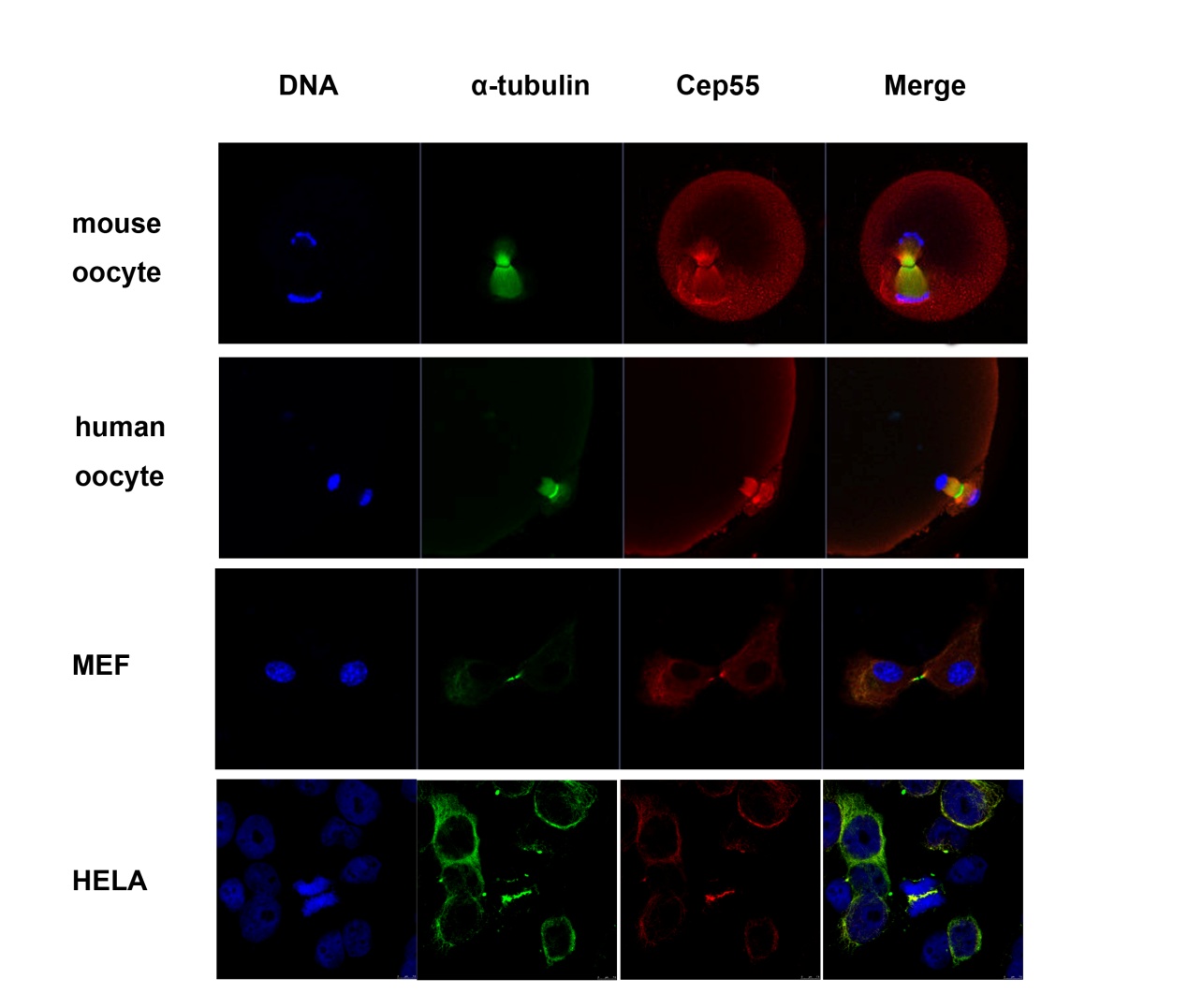


Supplementary figure1. Cep55 localization in different types of cells. To further validate the Cep55 antibody, mouse oocytes, human oocytes, mouse embryo fibroblasts and Hela cells in telophase were used to show Cep55 protein localization. Cep55 signal was localized to the mid-zone in only 2 of 57 Hela cells and no signal of Cep55 was detected in the 3 other types of cells. Oocytes and cells were stained for α-tubulin (green), Cep55 (red) and DNA (blue).

Supplementary video1,2,. live cell imaging of Cep55 localization. mRNA encoding fusion protein of Cep55 and GFP (green) was injected into the oocytes to trace the dynamics of Cep55 from MI to telophase I stages. Hoechst 33342 (red) was added to the culture medium 15 min before the oocytes were placed on the microscope stage.
